# Pediatric eosinophilic esophagitis effectively treated with a short-term 6-food-group elimination diet and reintroduction therapy: A case report

**DOI:** 10.1097/MD.0000000000016243

**Published:** 2019-06-28

**Authors:** Toshihiko Kakiuchi, Aiko Nakayama, Muneaki Matsuo

**Affiliations:** Department of Pediatrics, Faculty of Medicine, Saga University, Saga, Japan.

**Keywords:** 6-food-group elimination diet, eosinophilic esophagitis, reintroduction therapy, short-term

## Abstract

**Rationale:**

Eosinophilic esophagitis (EoE) is an inflammatory disease diagnosed based on clinical symptoms and pathological findings. EoE is treated with proton pump inhibitors (PPIs), topical steroids, and elimination diet-reintroduction therapy. After remission is achieved with the elimination diet, foods can be reintroduced sequentially to identify specific food triggers; however, this reintroduction method was not previously standardized.

**Patient concerns:**

A 12-year-old girl presented to our hospital with a 3-year history of epigastric pain. Esophagogastroduodenoscopy revealed linear furrows, esophageal rings, white exudates, and pallor throughout the esophagus. Histopathological findings revealed eosinophilic infiltration >15 eos/hpf on esophageal biopsy. There were no obvious abnormal findings in the stomach and duodenum.

**Diagnoses:**

EoE

**Interventions and outcomes:**

Because PPI was ineffective, we proposed a 6-food-group elimination diet (SFGED) and reintroduction therapy for EoE, which was initially planned out over a 6-week interval. However, a 5-day interval of SFGED and reintroduction therapy was performed instead. The treatment was effective and causative food antigens (egg and nuts) were identified. Since her symptoms recovered following short-term treatment, the nutritional impact was minimized, as was the duration of her hospitalization. Consequently, the patient's quality of life was well-preserved.

**Lessons:**

SFGED and reintroduction therapy for EoE may be effective even for short-term treatments involving 5-day intervals.

## 1 Introduction

Eosinophilic esophagitis (EoE) is an inflammatory disease, diagnosed based on clinical symptoms and pathological findings. Its symptoms result from esophageal narrowing and dysfunction due to the high and dominant infiltration of eosinophils into the esophageal mucosal epithelium.^[1,2]^ For the treatment of EoE, proton pump inhibitors (PPIs), topical steroids, and elimination diet therapies were used.^[3]^ Elimination diet therapy has not been established as a treatment for eosinophilic gastroenteritis,^[4,5]^ but for patients with EoE, it is proven effective, even in children.^[6,7]^ After remission is achieved with the elimination diet, foods can be reintroduced sequentially to identify specific food triggers, but this reintroduction was not previously standardized.^[8]^

Here, we report a case of a patient with EoE who was successfully treated with superior short-term 6-food-group elimination diet (SFGED) and reintroduction therapy.

## 2 Case presentation

A 12-year-old girl presented to our hospital with a 3-year history of epigastric pain that appeared regardless of meals. Antacids and intestinal agents were ineffective for symptom management. For 3 years, the cause of her weight loss and growth disorder attenuated, and symptoms of nausea, vomiting, and diarrhea were not observed. She exhibited asthma symptoms when the seasons changed, but no treatment was required. On her initial visit to our hospital, her vital signs were normal; body temperature was 36.5°C, heart rate was 65 beats per minute, and blood pressure was 102/62 mm Hg. A physical examination revealed only mild epigastric tenderness. Her height was 157.5 cm (+0.8 SD) and weight was 40.7 kg (−0.6 SD).

Her white blood cells count was 7600/μL (normal range: 3300–8600) and eosinophil percentage increased to 12% (<5.6). C-reactive protein (0.03 mg/dL; <0.14), erythrocyte sedimentation rate (3.0 mm/h; 3.0–15.0), and serum amyloid A protein (1.5 μg/dL; <8.0) were normal. Total immunoglobulin E (IgE) antibody was increased to 532 IU/mL (<170). The patient's antigen-specific IgE antibody test results are shown in Table 1. Stool bacterial culture test did not detect pathological bacteria, and eosinophils in feces were negative.

**Table 1:**
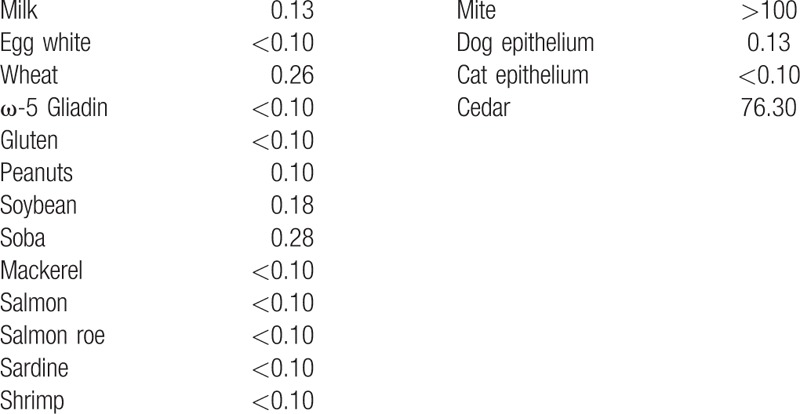
The values of antigen-specific immunoglobulin E before 6-food-group elimination diet therapy.

**Figure 1:**
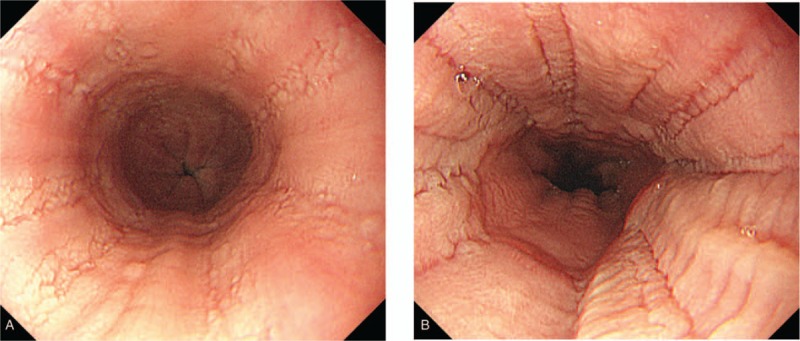
Esophagogastroduodenoscopy (EGD) shows linear furrows, esophageal rings, white plagues, and pallor throughout the esophagus. A, EGD findings of the lower esophagus. B, EGD findings of the middle and lower esophagus.

**Figure 2:**
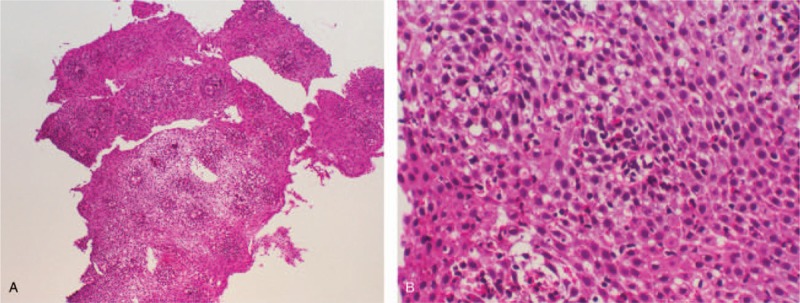
Histopathological findings show hypertrophy and papilla formation in the stratified squamous epithelial layer and deformation, regeneration, and infiltration of eosinophils in the basal layer. Esophageal biopsy reveals eosinophilic infiltration >15 eos/hpf (hematoxylin and eosin stain 200×).

To clarify the cause of her epigastric pain, we performed an esophagogastroduodenoscopy (EGD) which revealed linear furrows, esophageal rings, white exudates, and pallor throughout the esophagus (Fig. 1 A and B). There were no obvious abnormalities within the stomach or duodenum. Lower esophageal biopsy specimens revealed hypertrophy and papilla formation in the stratified squamous epithelial layer. In the basal layer, we noted conspicuous deformation, regeneration, and infiltration of eosinophils (Fig. 2 A and B). Contrast computed tomography examination of the chest and abdomen revealed no abnormalities. Notably, we observed no thickening of the esophageal wall. Ultimately, she was diagnosed with EoE based on EGD findings and pathological results.^[1,2]^

The patient commenced oral administration of PPI (esomeprazole 20 mg) for 8 weeks, with no improvement in epigastric pain. We suggested oral fluticasone propionate treatment, but the patient's parents expressed concern over steroid side effects. Therefore, we proposed an SFGED and reintroduction therapy for EoE.^[8]^ Owing to our inability to identify the suspected food by interview and no positive foods were identified with antigen-specific IgE antibody testing, SFGED was selected.

**Figure 3:**
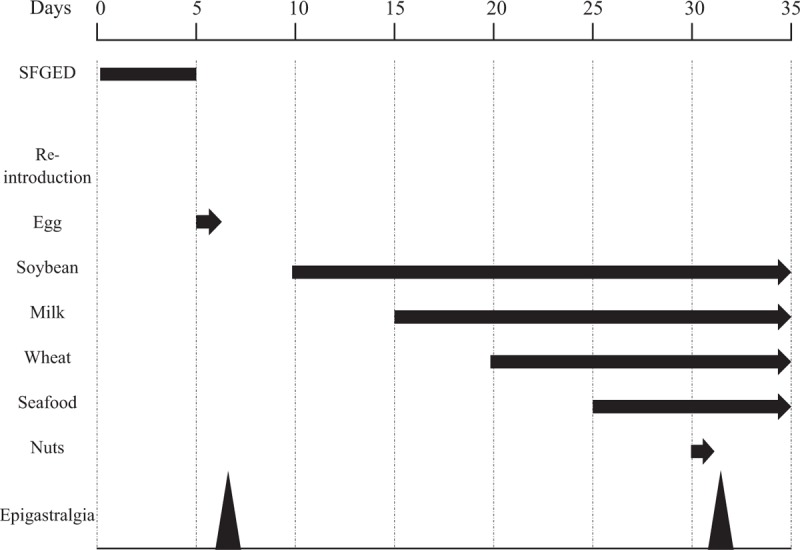
The patient's clinical course. SFGED and reintroduction therapy were performed at 5-day intervals. Epigastric pain appeared immediately after the patient consumed eggs and nuts, but her abdominal symptoms were relieved shortly after discontinuation of these foods. SFGED = 6-food-group elimination diet.

Initially, we planned to perform SFGED and reintroduction therapy at 6- and 2-week intervals, respectively.^[8]^ The 6 eliminated foods were eggs, soybeans, milk, wheat, seafood, and nuts. Figure 3 shows her clinical course. At the time of admission, she exhibited persistent epigastric pain; however, the pain completely disappeared immediately after starting SFGED, and her symptoms abated for 4 days thereafter.

According to the initial schedule, SFGED was planned for 6 weeks, but as her symptoms disappeared, we determined SFGED to be effective and shifted toward reintroduction therapy. Eggs were reintroduced first. The patient consumed an egg dish at breakfast and lunch, resulting in reemergence of intolerable nausea and epigastric pain appeared 6 hours after breakfast. We immediately eliminated eggs, and her abdominal symptoms disappeared completely the following morning. After stopping egg dishes, we noticed no recurrence of abdominal symptoms.

Next, we eliminated soybeans. Even following 5 days of observation, no abdominal symptoms appeared. We originally planned reintroduction therapy at 2-week intervals, but no symptoms appeared and we therefore changed to a 5-day interval. Accordingly, milk, wheat, and seafood were reintroduced, and abdominal symptoms were observed, but during the 5 hours of observation, no abdominal symptoms appeared. Finally, unbearable epigastric pain appeared 6 hours after adding nuts 1 time. We subsequently completely eliminated nuts. The next morning her abdominal symptoms disappeared.

As a result of reintroduction therapy, we determined that eggs and nuts caused our patient's EoE, and these foods were completely eliminated. Six months have passed since that decision, during which the patient has been free of epigastric pain, urticaria, and anaphylactic symptoms suggestive of food allergies.

## 3 Discussion

Our patient's disease course provided important clinical information. SFGED and reintroduction therapy for EoE may be effective even in short-term trials of 5-day intervals.

Elimination diet therapies for EoE are usually performed for 6 to 8 weeks,^[8]^ but long-term diet therapy can lead to malnutrition and adversely affect quality of life. If SFGED can be carried out over a shorter period of time, malnutrition risk can be minimized. In cases in which patients are hospitalized, accelerated SFGED may shorten the hospitalization period, potentially resulting in cost savings. During SFGED implementation, patients must tolerate a restrictive diet, so if elimination can be carried out over a shorter period of time, patients’ tolerance and compliance may be maximized. Our patient's abdominal symptoms disappeared completely 24 hours after starting the SFGED, and her symptoms did not recur for 4 days thereafter. Depending on the case, the duration of SFGED could be considerably shortened.

An elimination diet therapy for EoE involves the initial use of a 2-food-group elimination diet. If symptoms continue, treatment shifts to a 4-food-group elimination diet and SFGED. Reportedly, once the treatment period is shortened, the number of endoscopic examinations is also decreased.^[9,10]^ This elimination diet is equivalent to the plan we followed; however, it would take 2 to 3 times longer.

Unlike IgE-mediated food allergies, the symptoms of non-IgE-mediated food hypersensitivities are typically delayed for hours to weeks after ingestion of the culprit food.^[11]^ During our reintroduction therapy, there was a possibility exposures over our 5-day interval were unable to completely capture the non-IgE response. Foods other than eggs and nuts, judged not to be causative food agents over the 5 days, did not induce abdominal symptoms, even after continued ingestion. Original recommendations include a 2-week observation period after adding food^[8]^; however, causative foods can be observed during this abbreviated period. In this case, we were unable to carry out EGD after SFGED and introduction therapy without consent from the patient and her parents; consequently, imaging and pathological examinations were not performed. Six months after the diagnosis, which resulted in the removal of 2 kinds of food, our patient experienced no epigastric pain recurrence. These results suggest our reintroduction therapy method as beneficial for EoE.

In conclusion, SFGED and reintroduction therapy for EoE may be effective even when performed over a short-term 5-day interval. Future studies should examine additional cases of short-term elimination and reintroduction therapy for EoE.

## Acknowledgments

The authors would like to thank the patient and her family for consenting and allowing us to write and publish this case report.

## Author contributions

As the lead author, T.K. was involved with all stages of patient management and wrote the manuscript. T.K. and A.N. performed the treatment. M.M. collaborated as the reviewer. All authors read and approved the final manuscript.

**Conceptualization:** Toshihiko Kakiuchi.

**Data curation:** Toshihiko Kakiuchi, Aiko Nakayama.

**Formal analysis:** Toshihiko Kakiuchi.

**Investigation:** Toshihiko Kakiuchi, Aiko Nakayama, Muneaki Matsuo.

**Methodology:** Toshihiko Kakiuchi.

**Project administration:** Muneaki Matsuo.

**Supervision:** Muneaki Matsuo.

**Validation:** Toshihiko Kakiuchi.

**Writing – original draft:** Toshihiko Kakiuchi, Aiko Nakayama.

**Writing – review and editing:** Muneaki Matsuo.
